# Understanding the motivations for keeping wild birds in the semi-arid region of Brazil

**DOI:** 10.1186/s13002-018-0243-6

**Published:** 2018-06-11

**Authors:** Wallisson Sylas Luna de Oliveira, Sérgio de Faria Lopes, Rômulo Romeu Nóbrega Alves

**Affiliations:** 1 Programa de Pós-Graduação em Ciências Biológicas (Zoologia), Departamento de Sistemática e Ecologia, Universidade Federal da Paraíba, Cidade Universitária, João Pessoa, PB 58059-970 Brazil; 2 Departamento de Biologia, Universidade Estadual da Paraíba, Avenida das Baraúnas, 351, Campus Universitário, Bodocongó, Campina Grande, PB 58109-753 Brazil

**Keywords:** Songbird, Pets, Caatinga, Ethnoornitology, Ethnozoology

## Abstract

**Background:**

Birds are kept as pets around the world, and bird-keeping is an ancient and widespread practice, constituting one of the main reasons for the decline of some species. In the semi-arid region of Brazil, this practice is very common and continues despite being designated as illegal in recent decades.

This study aimed to identify the species and families of songbirds used as pets in the semi-arid region of Brazil, characterize the maintenance of the exploited species in captivity, and evaluate the sociocultural context associated with this practice.

**Methods:**

Data were collected from a total of 62 wild bird-keepers in the study area through interviews using semi-structured forms and informal conversations.

**Results:**

A total of 34 bird species are bred as pets in the study area. Thraupidae was the most represented family in this study followed by Icteridae, and together, these families accounted for 61.7% of the local specimens. As reported by the respondents, birds are acquired by capturing them in rural areas or through local and regional markets. The number of species identified by the respondents did not differ according to respondent income, educational level, or age (*p* > 0.05). Maintaining these birds in cages includes some care, such as providing feed, medicine, and in some cases, training to improve their song or to learn songs from other species. The species with the highest use values (UVs) were *Sporophila albogularis* (UV = 0.83), *Paroaria dominicana* (0.82), and *Sporophila nigricollis* (0.79), indicating their importance as wild animal pets.

**Conclusion:**

The birds reported in this study have strong cultural importance and high economic value for the people involved in bird-keeping. In this sense, ethnoornithological studies are fundamentally important since they can provide basic information to inform plans and actions to promote the conservation and sustainable management of local avifauna, including the essential element of environmental education strategies.

## Background

Birds are kept as pets around the world [1–5], and bird-keeping is an ancient and widespread tradition. However, this practice is considered one of the main reasons for the population declines of many species [6–10]. In particular, species of the order Passeriformes are kept as pets in cages, which is motivated by their distinct characteristics compared to other groups, including beautiful plumage and/or melodious singing [11].

Due to its large size and impressive biodiversity, Brazil has one of the most diverse avifauna in the world [12], including 1840 known bird species [13]. Considered the third largest biome in Brazil [14], the Caatinga encompasses most of the northeast region and houses, among other vertebrates, 591 birds’ species [15]. In the northeast of the country, maintaining birds in captivity is a common practice driven by the accessibility to several species that are kept as pets, especially passerines [10, 16]. In the semi-arid region of northeastern Brazil, keeping wild animals in captivity is as old as human occupation, and most ethnozoological research shows that birds are the most exploited group in the region, which is a major threat to the populations of many local species [1, 10, 17–19]*.* In this region (the Caatinga biome), birds are used for different purposes and have major social, economic, and cultural importance [1]. Several species of passerines and parrots are kept by local residents for pleasure, companionship, and ornamentation [10]. Keeping songbirds in cages, in both rural communities and urban areas [10, 12, 20–22], and the associated illegal trade has been identified as a major cause of the reduced population sizes of various species in Brazil [10, 20, 22–30].

Most of the research on the keeping and trade of wild birds is generally concentrated on points of sale, especially free-trade fairs. These studies involve animal apprehension by supervisory agencies including a wide range of wild bird taxa, with little detailed information on the capture and captive management of Passeriformes. This order includes songbirds and deserves special attention since it is the most exploited by clandestine commerce [23, 30–33].

In the municipality in which the present study was developed, it is common for songbirds to be captured and sold as pets to residents of both rural and urban areas, and this is illustrative of the situation in other municipalities of the Brazilian semi-arid region. In this context, the objective of this study was to identify the species and families of songbirds targeted by merchants and bird-keepers and to characterize the captive maintenance of these exploited species to answer the following questions. Is the number of species used locally associated with the age, income, or level of education of the respondents? Which factors influence the captive breeding of songbirds’ species in the surveyed region? Do endemic species tend to be better known and more commonly used by the respondents? What is the conservation status of locally exploited species?

## Methods

### Study area

**Fig. 1:**
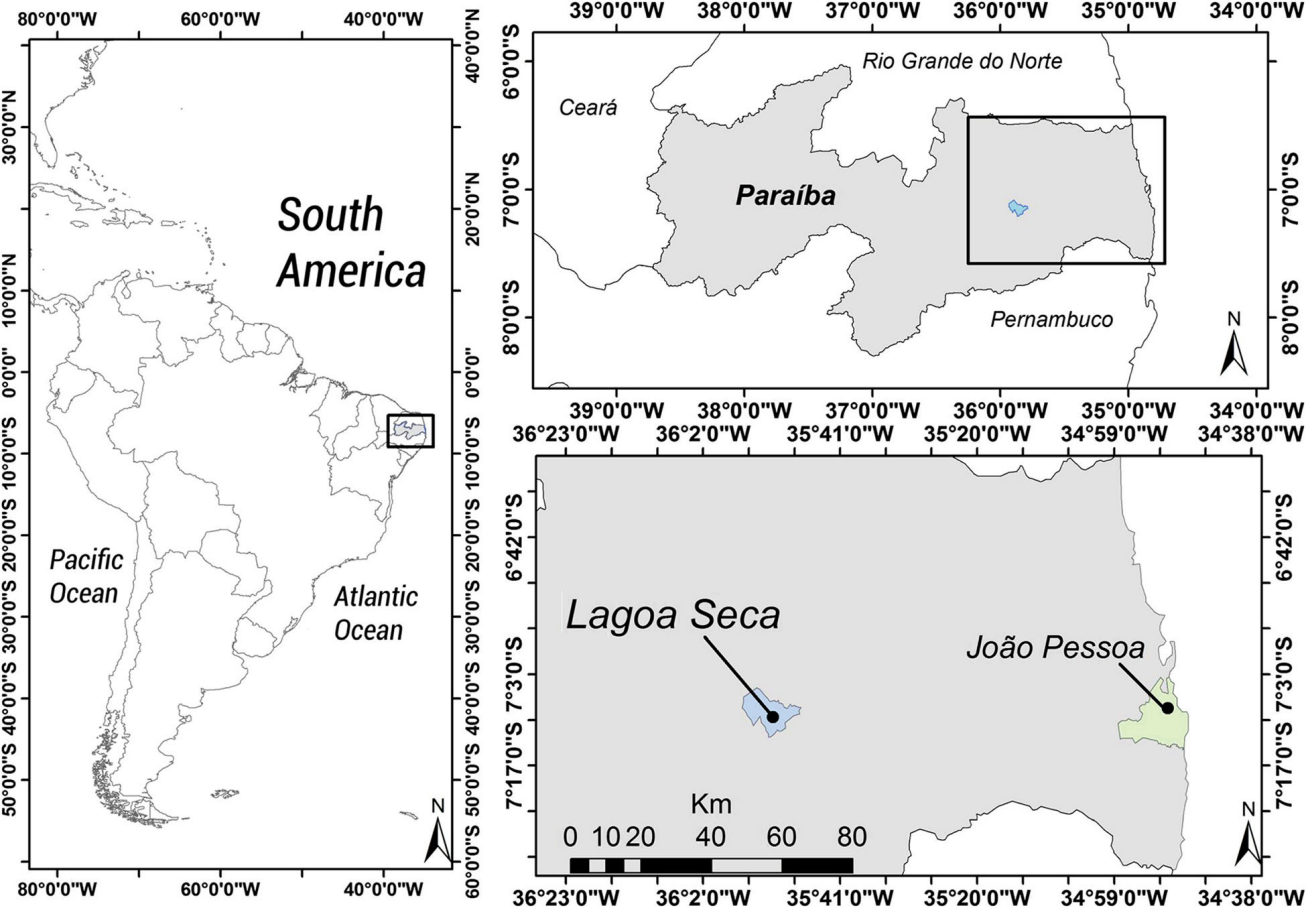
Location of the municipality of Lagoa Seca (07**°** 10′ 15″ S; 35**°** 51′ 14″ W) in the Agreste Mesoregion of Paraíba State (PB)

The study was conducted in the municipality of Lagoa Seca (07° 10′ 15″ S latitude; 35° 51′ 14″ W longitude) in the State of Paraíba, Brazil (Fig. 1). The municipality has an area of 107,589 km^2^ and a population of 25,900 inhabitants, of which 10,570 are urban and 15,330 are rural dwellers [34], and its human development index (HDI) is 0.627, according to the Human Development Atlas [35]. Lagoa Seca is in the Agreste Paraibano Mesoregion and the Borborema Plateau geo-environmental unit, and it is 109.4 km from the state capital of João Pessoa, Paraíba, Brazil. The vegetation in this unit is composed of sub-deciduous and deciduous forests that are typical of wilderness areas [36]. The climate is rainy tropical hot and humid, i.e., class A, AS’ (Köppen classification). The main economic activities in the municipality are trade and agriculture.

### Procedures

#### Data collection

Information was obtained by bird-keepers and bird-sellers from urban and rural Lagoa Seca through opportunistic visits from October 2015 to March 2017. Initially, the goal was to build trust with the first respondents through informal conversations, in which the nature and objectives of the research were explained, and consent to record the information was requested. Second, data on bird use were collected through semi-structured interviews supplemented by free-form interviews and informal conversations [37, 38]. The forms used in the interviews were designed to collect information on socioeconomic aspects (income, education, profession, and housing), the frequency and motives for bird use, the abundance of species in the region (very low, low, medium, and high), and information on the commercial aspects of bird-keeping. The socioeconomic data (income, age, and level of education) of the respondents are summarized in Table 1.

**Table 1:** Socioeconomic profile of the respondents in the municipality of Lagoa Seca, Paraíba, Brazil

Socioeconomic parameters	Number of respondents
Sex	
Female	2
Male	60
Age	
≤ 29	27
30–39	14
40–49	6
50–59	11
60–69	3
≥ 70	1
Marital status	
Single	24
Married	26
Stable relationship	8
Separated/divorced	2
Widow(er)	2
Profession	
Mason	7
Painter	1
Farmer	21
Public servant	1
Business and services	17
Taxi driver	2
Homemaker	2
Student	5
No profession	6
Monthly income (US$)	
≤ 167.00	4
167.00 to 300.00	19
≥ 300.00	23
No response	11
No stable income	5
Education	
Illiterate	2
Incomplete primary education	44
Complete primary education	3
Incomplete secondary education	1
Complete secondary education	10
Incomplete higher education	1
Complete higher education	1
Residence time	
< 20 years	10
20 to 40 years	34
41 to 60 years	15
> 60 years	2
Housing	
Own	54
Lease	8

The forms also included questions regarding the training and maintenance of captive birds (bird-keeping period, cost estimates, and animal singing characteristics) and the best capture period during the year. Based on the first interviews, further respondents were selected using the “snowball” technique [39], by which the initial respondent indicates other respondents for the researcher to reach.

Much information was gathered from direct observations of non-member participants [40] in the capture, maintenance, purchase, and sale of wild birds among respondents as well as visits to free-trade fairs where birds are sold.

#### Species identification

The birds mentioned by the respondents were identified as follows: (1) direct observations in the houses of the respondents or commercial establishments; (2) photographic records during interviews; (3) the use of the checklist*-*interview technique [41], in which photographs of birds are shown to respondents; (4) orientation by taxonomists familiar with the local avifauna; and (5) from prior ethnoornithological research in the region [1, 18, 32, 42]. Following species identification, the scientific nomenclature followed the guidelines of the Brazilian Ornithological Records Committee [13]. The Brazilian List of Species Threatened with Extinction [43] and the International Union for Conservation of Nature (IUCN) Red List [44] were used to determine the conservation status of each species.

### Data analysis

#### Use value

The use value (UV) (adapted from Phillips et al. [45] by Rossato et al. [46]) was calculated to illustrate the relative importance of each species as a function of each of its uses and was calculated as UV = Σ*U*/*n*, where UV is the value of a species, *U* is the number of mentions per species, and *n* is the total number of respondents. The UV of each species is only based on the importance attributed by the respondents and does not depend on any valuation of the researcher [47, 48].

#### Analysis of wealth estimates

An incidence matrix of the type of interviewee (lines) by the type of species (columns) was created, assigning the value of 1 for each species mentioned by a respondent and 0 for unmentioned species. The accumulation curves, in which the *X*-axis corresponded to the number of individuals interviewed and the number of species used, were randomized 100 times, and the mean values were calculated using the program EstimateS (version 8.2) [49]. The Chao 1 and Jackknife 1 estimators were used to estimate the total number of species in an area from the sampled data.

Statistical analyses were performed to determine the relationships between socioeconomic factors (age, income, and education) and the richness of the species used by the respondents. To determine the relationship between age and education and the number of species used, Spearman correlations were performed for non-parametric data, and a Kruskal-Wallis (*H*) test was used to determine if income influenced species richness, adopting a 5% level of probability (*p* < 0.05) [50, 51]. All tests were performed using the program Paleontological Statistics (PAST 2.17c) [52].

## Results and discussion

### Estimated richness of captive wild bird species

Respondents mentioned a total of 34 wild bird species from two orders and 11 families used as pets in the study region. These animals were acquired through illegal trade and/or captured by respondents themselves. Such practice was not surprising since these birds are chosen by bird-keepers for their diverse colours and songs, ease of maintenance, and in some cases, ability to imitate human speech [53]. These factors have led to preferences among bird-keepers for wild birds, especially passerines [10, 54–56].

**Fig. 2:**
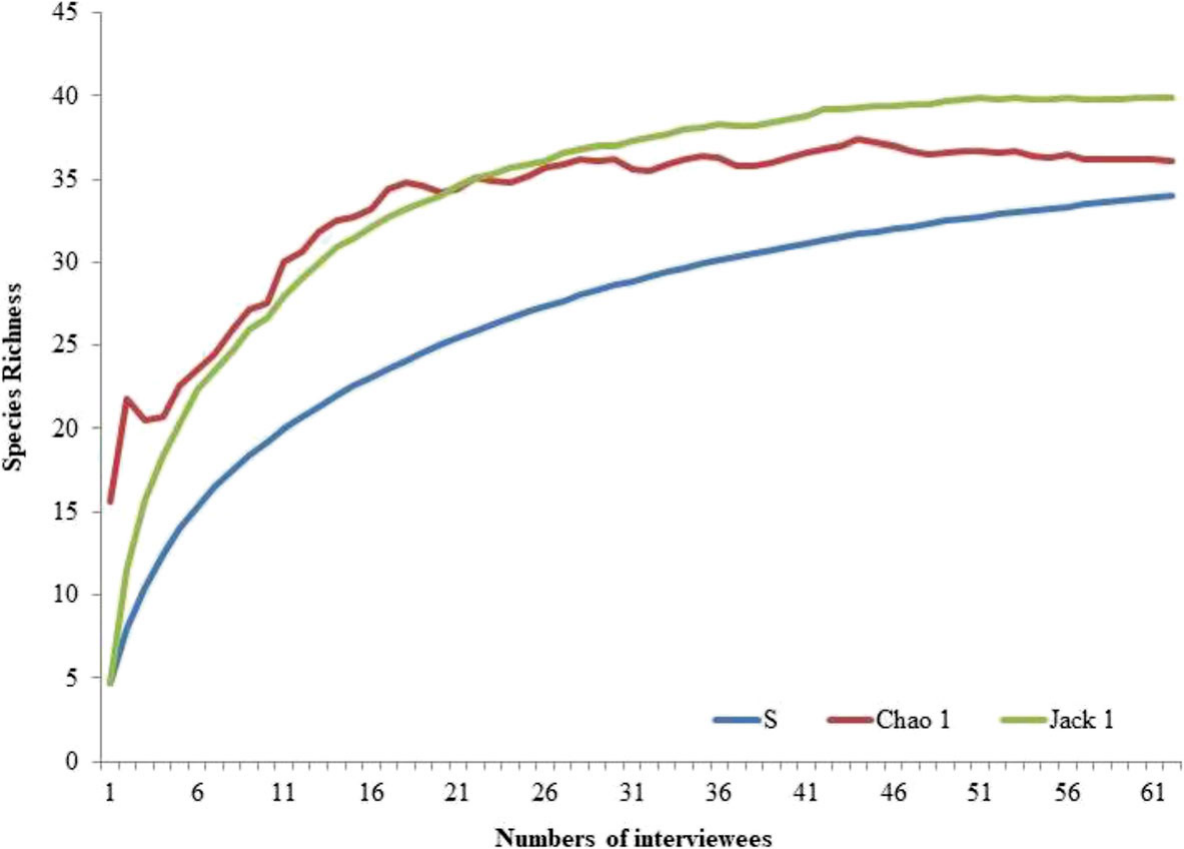
Rarefaction curves comparing the observed number of bird species (Sobs) with the estimated species richness in the studied region (Chao 1 and Jackknife 1). Calculated with 100 randomizations

The species richness recorded in the interviews (34 species) approximates that projected by the Chao 1 (36 species, 94.4%) and Jackknife 1 (39 species, 87.2%) estimators, demonstrating sample adequacy in relation to the number of interviews (Fig. 2). Regarding socioeconomic factors, no significant correlations were found between species richness and the age or education level of the respondents (*p* = 0.868 and *p* = 0.45, respectively). The Kruskal-Wallis test showed that the number of species mentioned did not vary according to income (*H* = 7.38, *p* = 0.111).

### Birds kept as pets

Because of the variety of species, their colourful plumage, small size, ease of maintenance in captivity, ease of transportation, and excellent singing capacity [10, 57, 58], Passeriformes are the most common birds in the world of illegal bird-keeping and trade, and these factors also govern the choice of bird species in the study area, located in the semi-arid region of Brazil, where bird-keeping is mainly associated with cultural issues, becoming a challenge from a sustainable and conservationist point of view [18, 59]. The necessity to consider the sociocultural context in conservation actions of wild birds on this region has been evidenced in recent ethnoornithological studies, which confirm the strong usage of birds for breeding and commercial purposes in several Brazilian semi-arid zones, although the legislation prohibits the use of wildlife in the country [17, 18, 23, 32, 33, 42, 60–66].

Of the 34 species mentioned by the respondents, Passeriformes was the order with the largest number of species (*n* = 32) followed by Psittaciformes, of which only two species were mentioned, the Turquoise-fronted Amazon *Amazona aestiva* and the Cactus Parakeet *Eupsittula cactorum* (Table 2). Of the mentioned species, 33 are native to Brazil, and six of those are endemic: the White-naped Jay *Cyanocorax cyanopogon*, the Campo Troupial *Icterus jamacaii*, Dubois’s Seedeater *Sporophila ardesiaca*, the White-throated Seedeater *Sporophila albogularis*, the Red-cowled Cardinal *Paroaria dominicana*, and the Cactus Parakeet *E. cactorum* (the last three are endemic to the Brazilian Caatinga). The species *Spinus yarrellii* (Yellow-faced Siskin) is listed on the Brazilian Ministry of the Environment (MMA) and the IUCN Red List as “Vulnerable” [44, 67] (Table 2). The only non-native bird, *Estrilda astrild* that is commonly known as the St. Helena Waxbill, is an introduced species, but because it was introduced to Brazil long ago and has adapted well, the Brazilian Institute of the Environment and Renewable Natural Resources (IBAMA) has chosen to treat it as a wild species, so its capture and trade is also prohibited [23]. Although few respondents (*n* = 34) reported keeping parrots as pets, those that did claimed that the demand for *E*. *cactorum* is very high in the region and that they acquired this species by purchase, including for resale, or received it as a gift. The Psittacidae family (which includes parrots, parakeets, and macaws) is strongly affected by global trade [4, 68–70]; it is the second most traded bird family in the world [8].

**Table 2:** List of wild bird species used in commerce and as pets in the municipality of Lagoa Seca, Paraíba, Brazil, including taxonomy, common name, number of mentions by use modality, use value (UV), and conservation status

Taxonomy	Common name	Citations by use modality		UV	Conservation status	
		Creation	Trade		IUCN	MMA
Order/family/species						
Passeriformes						
Thraupidae						
*Sporophila lineola* (Linnaeus, 1758)	Lined Seedeater	18	16	0.54	LC	LC
*Sporophila nigricollis* (Vieillot, 1823)	Yellow-bellied Seedeater	28	21	0.79	LC	LC
*Sporophila ardesiaca* (Dubois, 1894)	Dubois’s Seedeater	2	2	0.06	LC	LC
*Sporophila albogularis* (von Spix, 1825)	White-throated Seedeater	30	22	0.83	LC	LC
*Sicalis luteola* (Sparrman, 1789)	Grassland Yellow-finch	4	3	0.11	LC	LC
*Volatinia jacarina* (Linnaeus, 1766)	Blue-black Grassquit	7	6	0.20	LC	LC
*Sicalis flaveola* (Linnaeus, 1766)	Saffron Finch	16	10	0.41	LC	LC
*Tangara cayana* (Linnaeus, 1766)	Burnished-buff Tanager	5	5	0.16	LC	LC
*Tangara palmarum* (Wied, 1821)	Palm Tanager	4	4	0.12	LC	LC
*Tangara sayaca* (Linnaeus, 1766)	Sayaca Tanager	20	17	0.59	LC	LC
*Paroaria dominicana* (Linnaeus, 1758)	Red-cowled Cardinal	27	24	0.82	LC	LC
*Sporophila angolensis* (Linnaeus, 1766)	Chestnut-bellied Seed-Finch	1	1	0.03	LC	LC
*Sporophila bouvreuil* (Statius Müller, 1776)	Copper Seedeater	4	0	0.06	LC	LC
*Coryphospingus pileatus* (Wied, 1821)	Pileated Finch	3	1	0.06	LC	LC
*Saltator similis* d’Orbigny & Lafresnaye, 1837	Green-winged Saltator	2	2	0.06	LC	LC
*Sporophila leucoptera* (Vieillot, 1817)	White-bellied Seedeater	1	1	0.03	LC	LC
*Coereba flaveola* (Linnaeus, 1758)	Bananaquit	1	1	0.03	LC	LC
Icteridae						
*Icterus pyrrhopterus* (Vieillot, 1819)	Variable Oriole	4	2	0.09	LC	LC
*Icterus jamacaii* (Gmelin, 1788)	Campo Troupial	5	4	0.14	LC	LC
*Gnorimopsar chopi* (Vieillot, 1819)	Chopi Blackbird	2	2	0.06	LC	LC
*Chrysomus ruficapillus* (Vieillot, 1819)	Chestnut-capped Blackbird	1	0	0.01	LC	LC
Fringillidae						
*Spinus yarrellii* (Audubon, 1839)	Yellow-faced Siskin	7	6	0.21	VU	VU
*Euphonia chlorotica* (Linnaeus, 1766)	Purple-throated Euphonia	3	1	0.06	LC	LC
Turdidae						
*Turdus rufiventris* Vieillot, 1818	Rufous-bellied Thrush	15	14	0.46	LC	LC
*Turdus amaurochalinus* Cabanis, 1850	Creamy-bellied Thrush	1	1	0.03	LC	LC
*Turdus leucomelas* Vieillot, 1818	Pale-breasted Thrush	14	12	0.41	LC	LC
Mimidae						
*Mimus saturninus* (Lichtenstein, 1823)	Chalk-browed Mockingbird	4	4	0.12	LC	LC
Cardinalidae						
*Cyanoloxia brissonii* (Lichtenstein, 1823)	Ultramarine Grosbeak	23	22	0.72	LC	LC
Passerelidae						
*Zonotrichia capensis* (Statius Muller, 1776)	Rufous-collared Sparrow	26	21	0.75	LC	LC
Corvidae						
*Cyanocorax cyanopogon* (Wied, 1821)	White-naped Jay	1	1	0.03	LC	LC
Tyrannidae						
*Pitangus sulphuratus* (Linnaeus, 1766)	Great Kiskadee	2	2	0.06	LC	LC
Estrildidae						
*Estrilda astrild* (Linnaeus, 1758)	Common Waxbill	2	1	0.04	LC	LC
Psittaciformes						
Psittacidae						
*Eupsittula cactorum* (Kuhl, 1820)	Cactus Parakeet	2	1	0.04	LC	LC
*Amazona aestiva* (Linnaeus, 1758)	Turquoise-fronted Amazon	1	1	0.03	LC	LC

In the study region, the use of wild birds as pets is directly associated with trade. Most of the respondents (*n* = 47) stated that in addition to keeping the mentioned species as pets, they directly participated in their purchase and sale. However, only a few respondents (*n* = 15) said that they only keep birds as pets and that their participation in trade is limited to purchase at fairs in the adjacent municipality of Campina Grande, Paraíba, Brazil, or from local merchants.

**Fig. 3:**
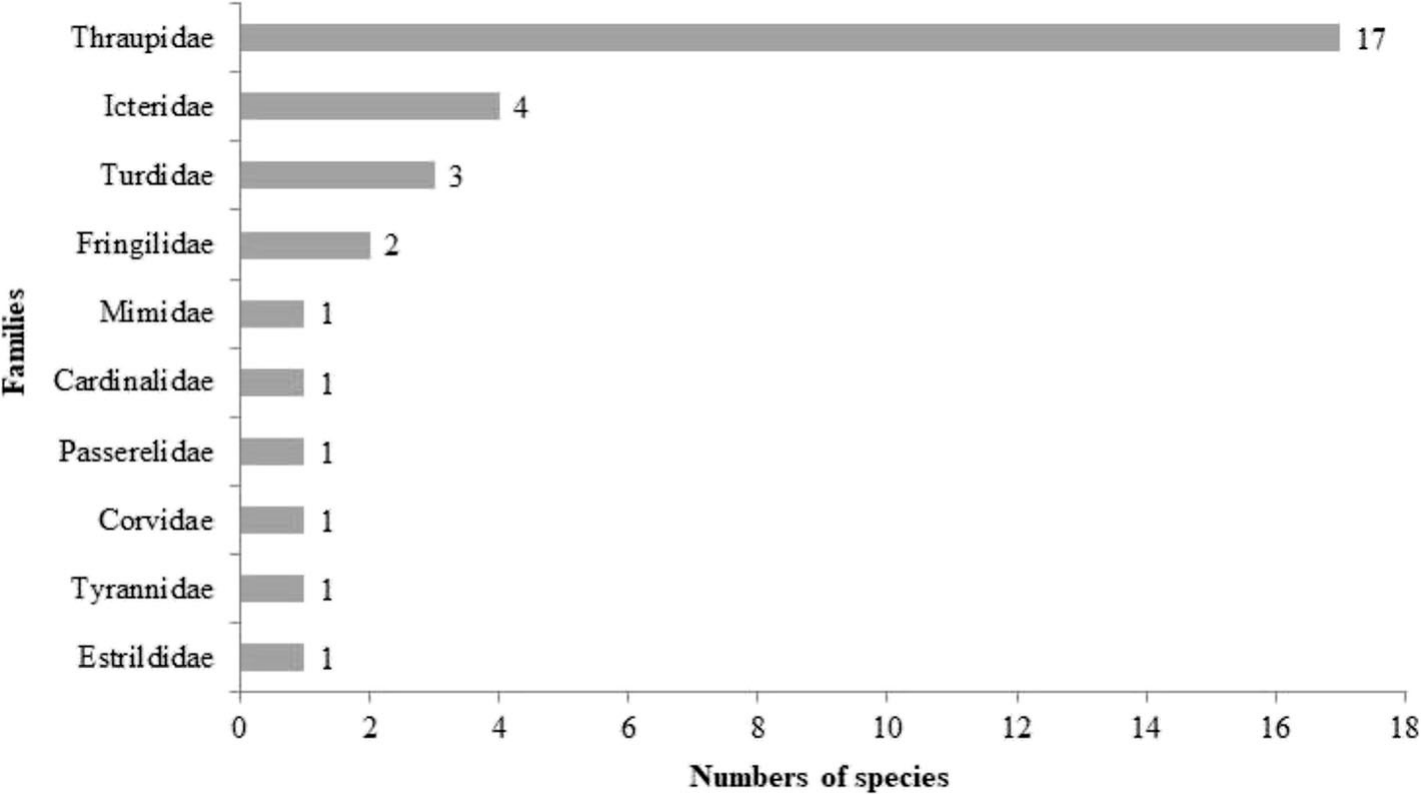
Representation of passeriform families and numbers of species observed in the municipality of Lagoa Seca, Paraíba, Brazil

The most common families mentioned by the respondents were Thraupidae, which corresponded to 61.7% of the locally used specimens, followed by Icteridae (Fig. 3). The preference for keeping species of these families as pets is often observed in other areas of Brazil [1, 18, 23, 32, 60, 71–73] and is likely because this family includes beautiful Brazilian birds with large vocal repertoires [74, 75]. In a study conducted on the avifauna seized and voluntarily delivered to the IBAMA Wild Animal Triage Centre in the city of Juiz de Fora in Minas Gerais State, Brazil, Gogliath et al. [31] found many specimens from the families Thraupidae, Icteridae, and Psittacidae, of which seedeaters (*Sporophila* sp.), Saffron Finch (*Sicalis flaveola*), Green-winged Saltator (*Saltator similis*), Chopi Blackbird (*Gnorimopsar chopi*), and White-eyed Parakeet (*Aratinga leucophthalma*) were the most frequently possessed by bird-keepers and bird-sellers. In the surveyed area, birds such as *Zonotrichia capensis* (Passerelidae), *Cyanoloxia brissonii* (Cardinalidae), and *Turdus* sp. (Turdidae) are highly sought after for cage breeding, as they stand out due to their beauty and song, besides being easily kept in captivity.

When asked which birds are the most difficult to find in the local environment, among the most mentioned were *S*. *yarrellii*, *Sporophila angolensis*, and *I*. *jamacaii*, and some respondents even stated that they had not seen *S*. *yarrellii* in the wild for a long time and that the species is currently rare in captivity. According to the respondents, there are several reasons for the local disappearance of these species including the deforestation of rural areas for agricultural production, pesticide application on legume seeds (which kills granivorous birds), and exploitation pressure for bird-keeping and bird-selling. Additionally, some respondents described cases where small farmers mix poison with corn bran and spread it over their crops to prevent birds from feeding on planted seeds, thus killing birds that feed on the poisoned bran.

### Main species and use value (UV)

**Fig. 4:**
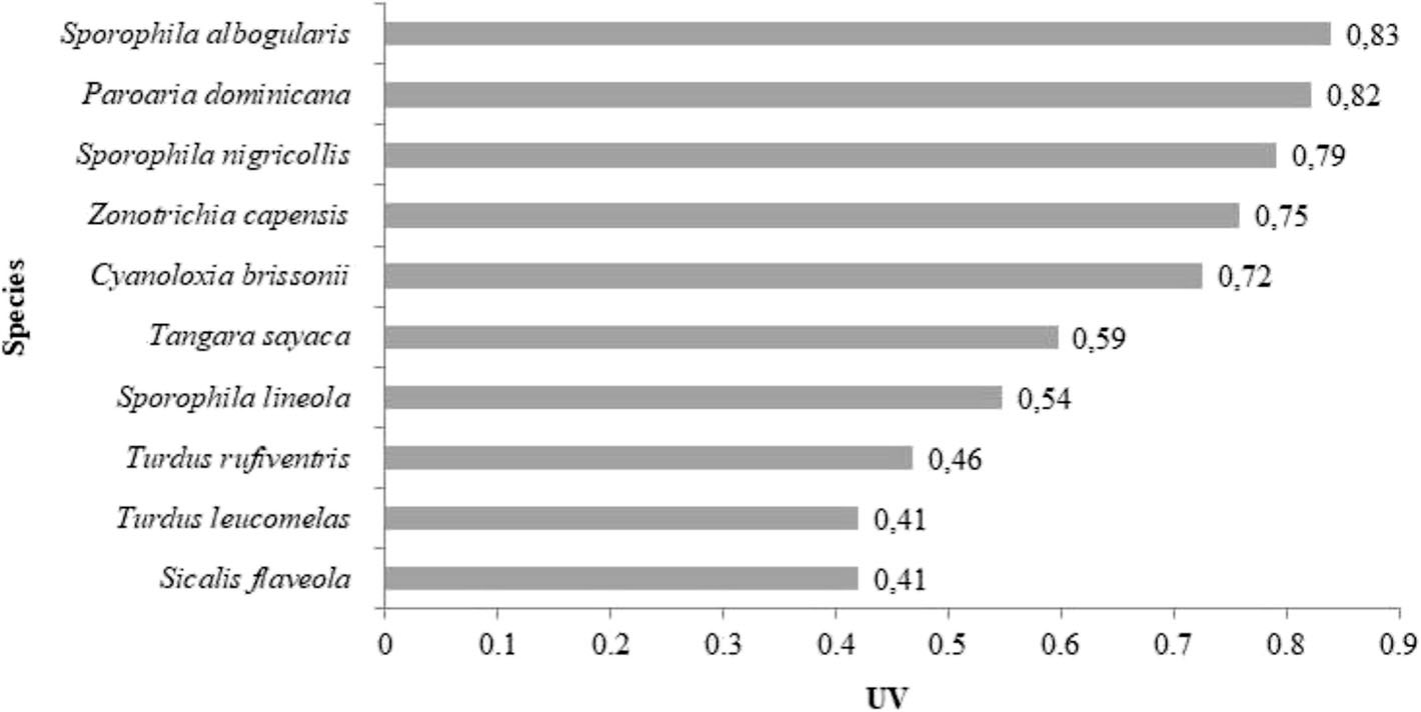
Main species reported by the respondents and their respective use values (UVs)

**Fig. 5:**
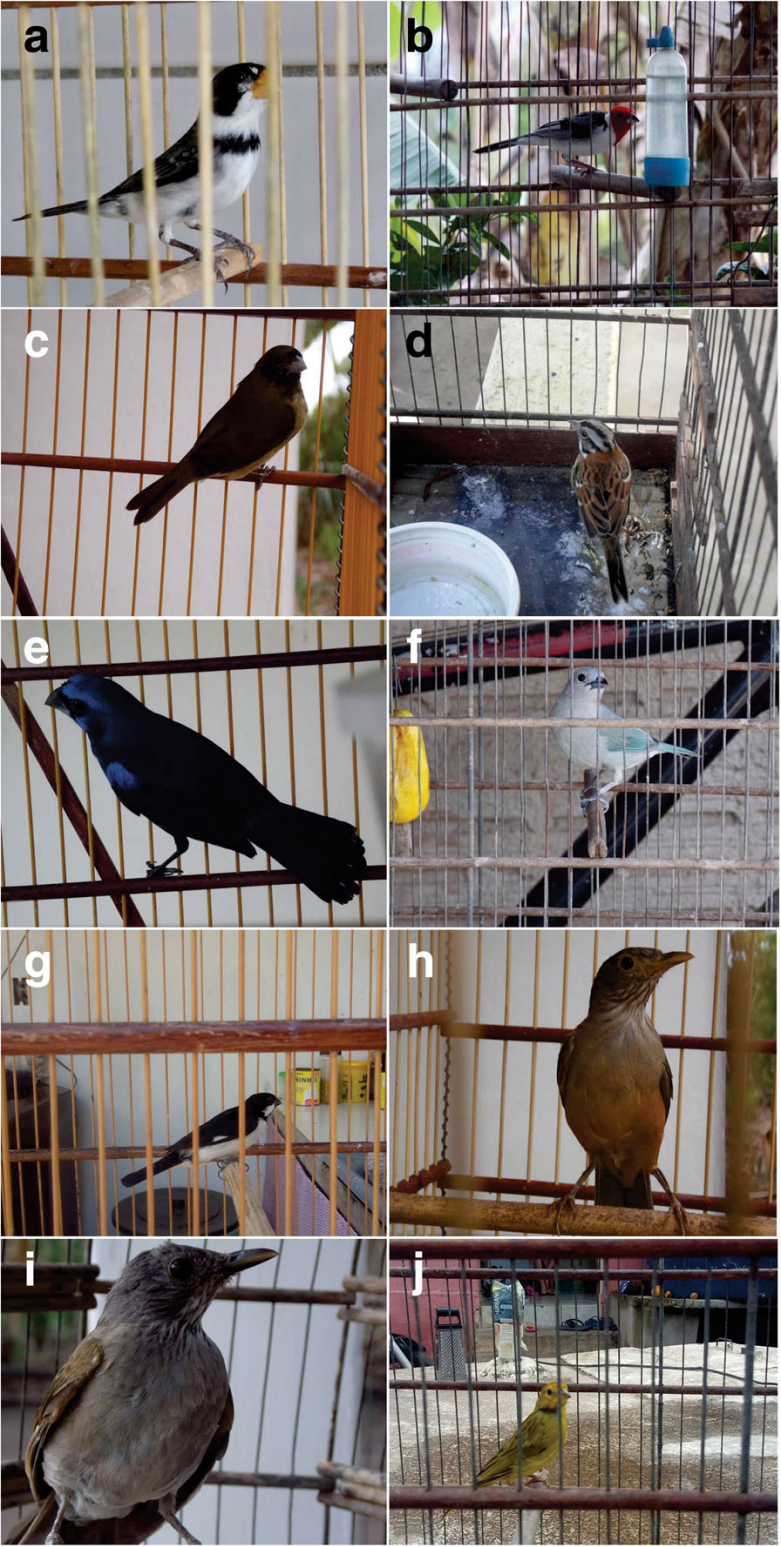
Main species of wild birds used in the study area: *Sporophila albogularis* (**a**), *Paroaria dominicana* (**b**), *Sporophila nigricollis* (**c**), *Zonotrichia capensis* (**d**), *Cyanoloxia brissonii* (**e**), *Tangara sayaca* (**f**), *Sporophila lineola* (**g**), *Turdus rufiventris* (**h**), *Turdus amaurochalinus* (**i**), and *Sicalis flaveola* (**j**). Photos: Wallisson Sylas Luna de Oliveira

The importance of each species reported in the present study for bird-keeping purposes is reflected in the respective UVs (Figs. 4 and 5), which varied between 0.01 and 0.83. The most important birds were *S*. *albogularis*, *P*. *dominicana*, *Sporophila nigricollis*, *Z*. *capensis*, *C*. *brissonii*, *Tangara sayaca*, *Sporophila lineola*, *Turdus rufiventris*, *Turdus leucomelas*, and *S*. *flaveola*.

Birds of the genus *Sporophila* are widely distributed throughout South America and are highly appreciated for their beautiful singing capacity [76, 77]. The prevalence of birds in this genus mentioned as being captured to be kept in cages (especially *S*. *albogularis*, a species endemic to the Caatinga with the highest UV in this study (UV = 0.83)) has also been highlighted in other studies of bird-keeping, selling, and seizure throughout Brazil [1, 10, 23, 33, 60, 61, 78–81].

Another species that stands out among the most mentioned birds in ethnoornithological studies in Northeast Brazil is *P*. *dominicana*, an endemic of the Caatinga biome [74] that is a favourite of bird-keepers and bird-sellers for various reasons, including easy capture [18, 19, 32, 59, 62, 63, 82]. It had the second highest UV in this study (UV = 0.82).

Species in the Thraupidae family were mentioned the most in the present study, as highlighted by their UVs. As already discussed, the preference for this family is because it includes beautiful Brazilian birds with beautiful singing capacity. In a study of wild bird seizures in the state of Amazonas over 20 years (1992–2011), Nascimento et al. [80] reported that the family Thraupidae accounted for the most species. Thus, Thraupidae species are commonly kept in various regions of Brazil [79, 81, 83].

### Acquisition and maintenance in cages

Respondents stated that they acquired their birds directly from the wild (capture) as well as from illegal trade at fairs or among local bird-keepers (most common practice) as well as those from other localities. This finding indicates the existence of a commercial network involving the municipality of Lagoa Seca and other municipalities.

According to the interviewees’ testimonies, birds are captured directly from nature using four techniques: “assaprão,” “visgo,” “arapuca,” and “redinha or assaprão de rede”, which are toward to different bird species, taking into account the size of the animal, food habit, and locations and times of the year for capture. Of the techniques used, the ones with greater citations were the assaprão (*n* = 35) and the visgo (*n* = 15). Detailed descriptions of these techniques can be consulted in previous studies [42, 66, 84]. These bird-catching techniques are widely used among breeders from various Brazilian semi-arid sites [18, 23, 33, 62, 64, 71, 84], making it possible to capture a large number of species used as pets.

Rural respondents capture birds throughout the year but prefer the rainy season because it is the breeding period for most birds when there is abundant food for many species, especially the genus *Sporophila*, members of which have been reported as caged birds in several studies in Brazil [1, 18, 33, 54, 61, 64, 85]. Among the species captured or sold for bird-keeping, males are the most sought after because they have greater singing capacity and more beautiful plumage than females. According to Ribeiro and Silva [22], the preference for male individuals has had a very large negative impact on the populations of target species because approximately 90% of bird species breed monogamously.

In addition to free markets and fairs, the purchase of songbirds for captivity breeding through illegal trade is also evidenced by obtaining and selling these animals at strategic sites, such as meeting places and in households of breeders and traders, in order to avoid attention of supervisory bodies, establishing a decentralized clandestine marketing network.

**Fig. 6:**
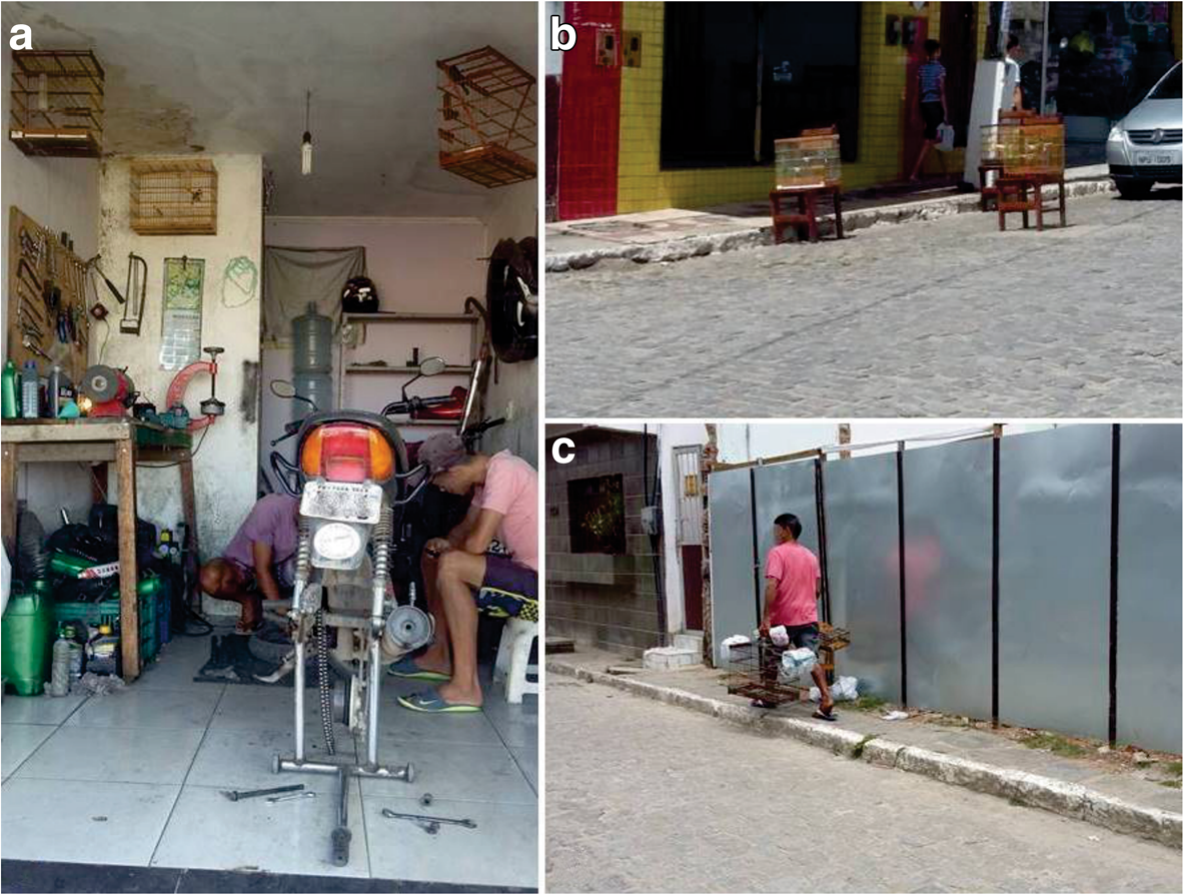
Exposed cages with wild birds in the streets and commercial establishments in the municipality of Lagoa Seca, Paraíba, Brazil. Motorcycle workshop (**a**), bar and restaurant (**b**), and a local bird-keeper returning to his home after catching songbirds (**c**). Photos: Wallisson Sylas Luna de Oliveira

Most bird-keepers use cages, but some respondents also mentioned using aviaries, which have more space but house multiple individuals of different species and sizes, which can cause stress and even result in fighting among birds. According to the respondents, larger birds are kept in larger cages. In the study area, it is very common to find cages hanging from the ceiling and in front of the homes of bird-keepers as well as in commercial establishments, and people walk the streets with cages in hand (Fig. 6).

**Fig. 7:**
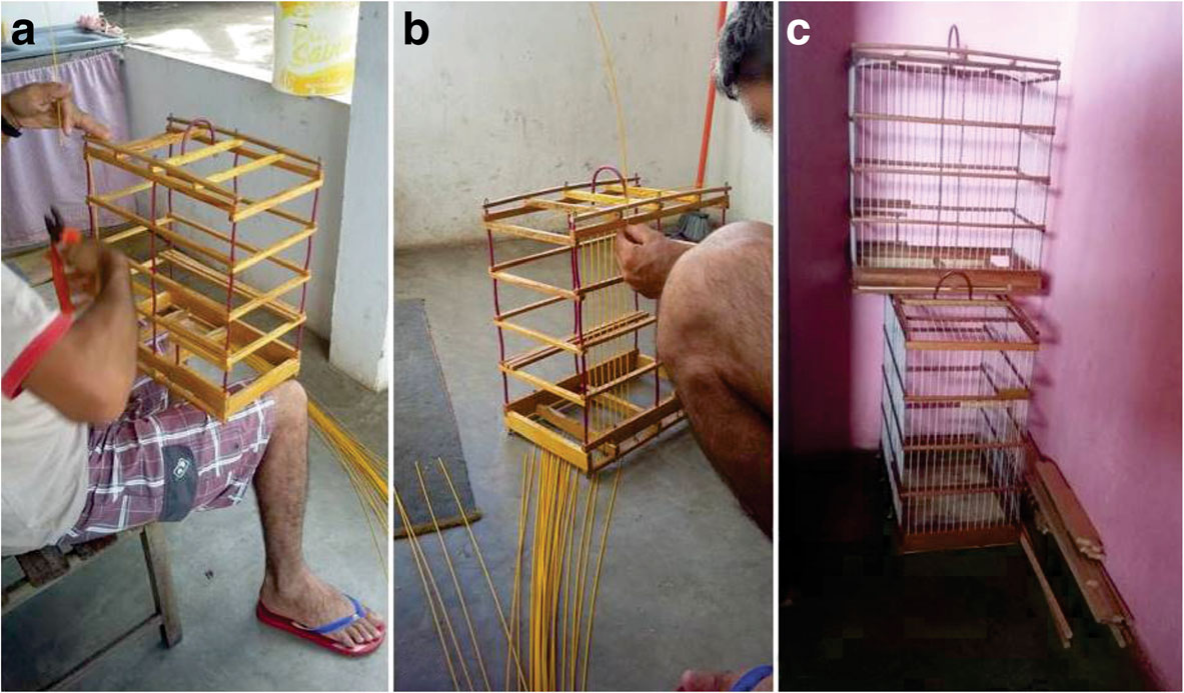
Production of cages by one of the respondents. Making cages from wood, wire, and fibre rods (**a**, **b**) and cages ready for use or for sale (**c**). Photos: Wallisson Sylas Luna de Oliveira

Cages are primarily crafted from wood and wire and designed to hold only one individual, except for Saffron Finches *S*. *flaveola*, which are locally known as “canários-de-briga” and whose cages are larger to accommodate pairs of birds in multiple compartments for fights. Some respondents claimed to produce cages for their own use as well as sale to other bird-keepers (Fig. 7), thus earning extra income and saving money when purchasing birds. The respondents further indicated that the quality of a cage is directly associated with the quality of a bird, that is, birds with more beautiful plumage and greater capacity for singing are housed in more beautiful cages.

In general, after songbirds are removed from their natural environment, they require management to adapt to a life in captivity, which requires a certain period of time. According to the respondents, newly captured birds are known as “bicho brabo” (wild beasts) because they are unused to captivity, do not sing, and struggle intensely inside the cage, sometimes injuring themselves. “Pássaro em ordem” (bird in order) is the local term for a bird that has become adapted to captivity and moulted and that sings well and often.

The ability to mimic other songbirds or song melodies is common among some species that are captured and sold locally, and it may strongly influence the commercial value of these species. For example, *S*. *albogularis* mimics the song of *C*. *brissonii*, and *I*. *jamacaii* mimics other species and musical melodies. To develop the song of a young *S*. *albogularis*, its cage is kept near that of a strong-singing adult *C*. *brissonii* until it faithfully reproduces the *C*. *brissonii* song; this training requires an average of 4 to 6 months. To develop the capacity for sound imitation in *I*. *jamacaii*, individuals of this species are kept in a room and forced to listen to CDs playing the preferred bird song or other music for hours. Similar training was observed by Gama and Sassi [23] in a study of aspects of the illegal commercialization of wild birds in the city of João Pessoa, Paraíba, Brazil, where newly captured or sold birds are exposed to recorded birdsong and a conspecific or heterospecific “teacher” bird.

**Fig. 8:**
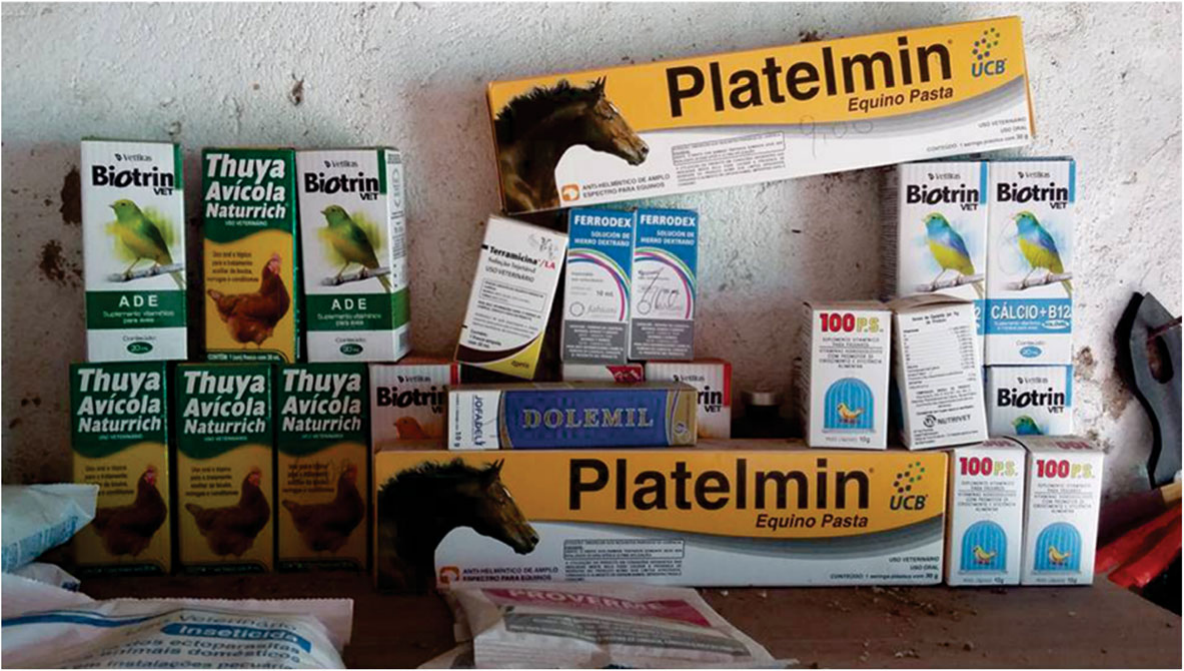
Bird medicine (a vitamin and calcium compound) sold in a feed store in the municipality of Lagoa Seca, Paraíba, Brazil. Photo: Wallisson Sylas Luna de Oliveira

Feeding is a crucial factor in the well-being and development of bird song and determines the success of captive management [86]. All respondents in the study area reported that both song and plumage quality are influenced by the diet of the bird, so the respondents offer a balanced diet based on fruit, feed, seed, millet, and birdseed that varies by species. In some cases, respondents reported giving vitamin compounds and calcium-based medicines to maintain the health, song, and plumage of the birds (Fig. 8).

Cage maintenance (sanitation and the provision of water and food) is normally performed once a day, but during the moulting season, when the bird undergoes a change in plumage, it does not sing for a long time and is vulnerable to disease, thus requiring extra care including limited exposure to wind and other birds. At this stage, medicines and vitamins are added to the diet whenever possible [23]. When questioned about food expenditures, respondents stated that the monthly cost is low, averaging $5.00 to $7.00 US since a 500-g package of birdseed, millet, or feed costs $1.00 US on average, but according to the respondents, this amount increases some when it is necessary to buy medicines and vitamin compounds, which can be bought in local feed stores along with feed.

**Fig. 9:**
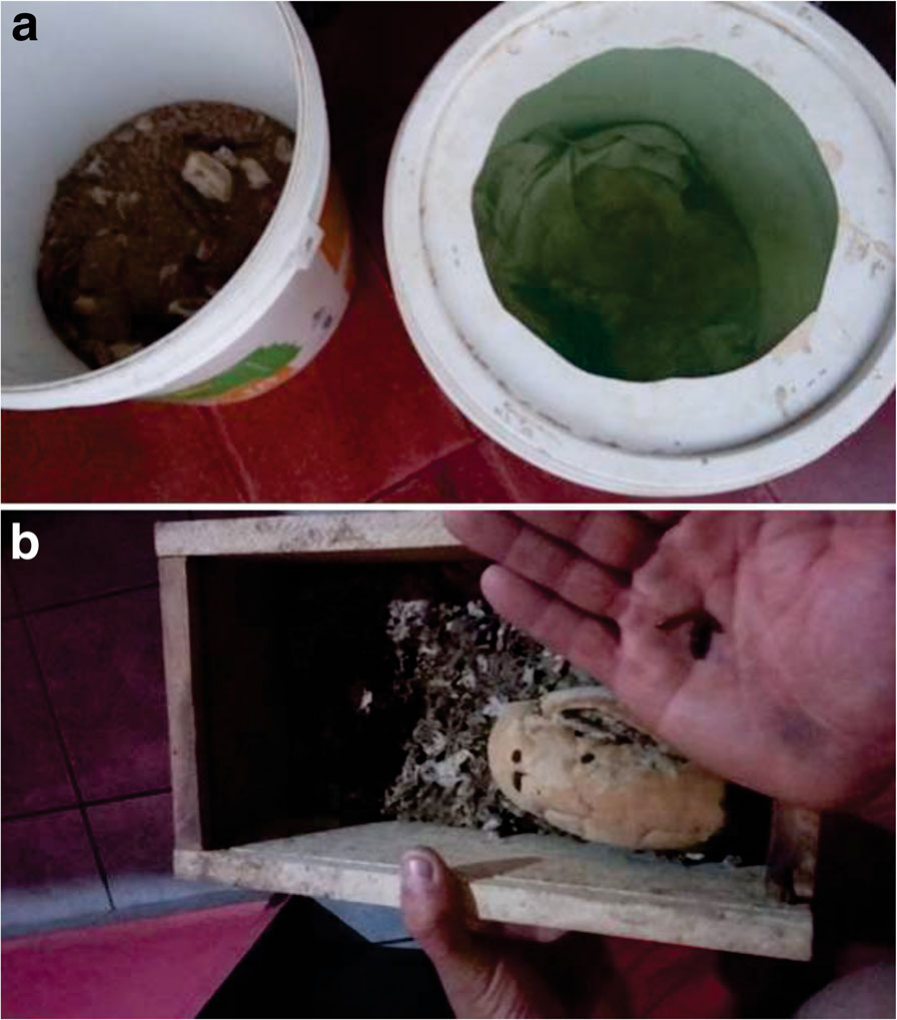
Feeding *Tenebrio molitor* (Linnaeus, 1758) with larvae cultivated by the respondents. Cultivation of larvae in buckets (**a**) and in small wooden crates (**b**). Photos: Wallisson Sylas Luna de Oliveira

In addition to this basic diet, some respondents reported using mealworms, *Tenebrio molitor* Linnaeus, 1758, locally known as “tenébrio” (Fig. 9), that the respondents rear in their homes. The larvae are used as a nutrient-rich food supplement that benefits the health and song quality of birds such as *S*. *albogularis*, *S*. *angolensis*, *Z*. *capensis*, *S*. *nigricollis*, *I*. *jamacaii*, *P*. *dominicana* (Linnaeus, 1758), *C*. *brissonii*, and *S*. *flaveola*.

### Bird fights: “rinhas”

**Fig. 10:**
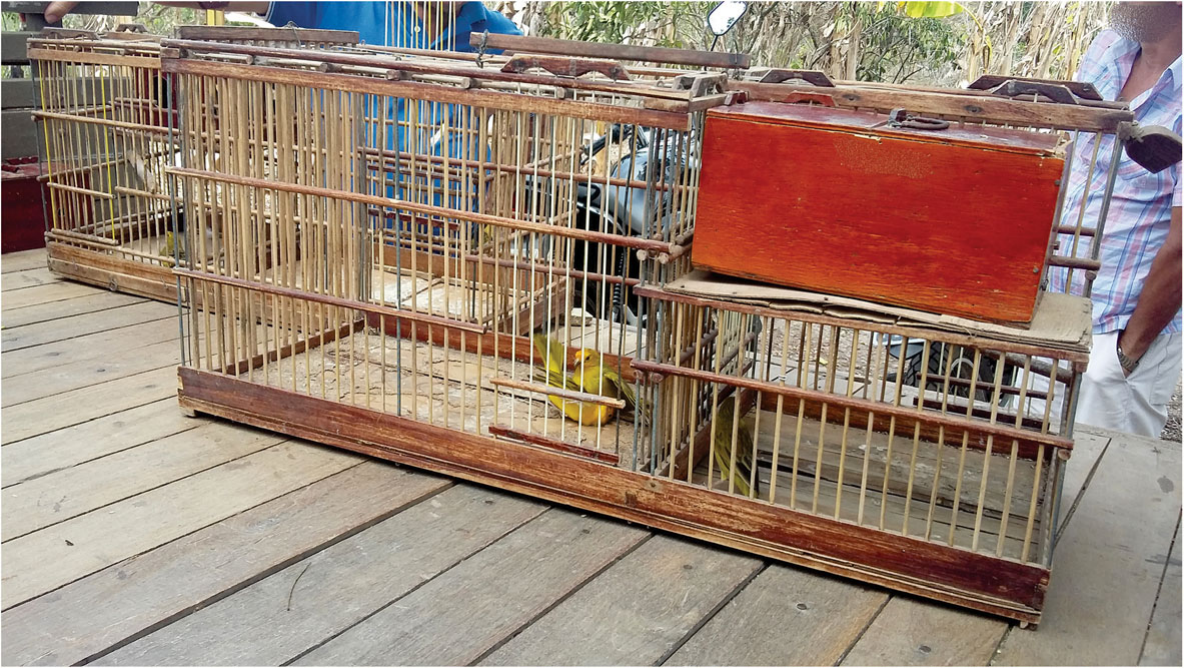
Record of a fight between Saffron Finches (*Sicalis flaveola*, Linnaeus, 1766) in the municipality of Lagoa Seca, Paraíba, Brazil. Photo: Wallisson Sylas Luna de Oliveira

In addition to trapping songbirds to keep them in cages as pets, certain species are commonly used in bird fights in the northeast and other parts of Brazil; this is an illegal practice known locally as “rinhas” [10, 18, 23, 87, 88]. The main species for this purpose is *S*. *flaveola*, but *C*. *brissonii* and *P*. *dominicana* are also used. In the present study, many respondents indicated knowledge of this practice in the region, but only four said they engaged in this activity with *S*. *flaveola*. Fighting occurs in the presence of bird owners and other people, who place bets on the bird they believe will win, and according to the respondents, bets range from $10.00 to $167.00 US. Fights occur inside cages that have several compartments (Fig. 10) including a “cumbuco,” which is a movable compartment coupled to the inside of a cage that can accommodate a pair of birds. According to the respondents, female birds are necessary to “rile up” the males for these fights. After pairing the cages, the movable compartments are opened, giving the males access to each other, and the females exit to another compartment but remain visible to their male partners. The fight only ends when one of the birds attempts to flee or is badly injured. The opponent is declared victorious, and the birds are separated by their owners.

### Trends and future perspectives

Keeping wild birds is an antique practice in Brazil [83], recorded in the first historical documents during the colonial period. This cultural activity has been persistent over time despite the prohibitions of environmental agencies in the last century [89], a situation that led this practice to occur clandestinely in a widespread way, especially in some regions of Brazil, as in the semi-arid Northeast. Our results reinforce this situation, revealing that bird-keeping has attracted the interest of many people, driving an illicit trade in wild birds. The issue is intensified by the lack of or little supervision by environmental agencies, and when birds become part of the illegal trade in region, which interconnects various cities and intensifies wild species exploitation as pets in surveyed area. As a result of this persistent illegal trade, associated to others problems as a habitat loss, many songbird species has become rare or endangered due to over-exploitation.

The future scenarios point to the clandestine maintenance of this practice in surveyed area and all other Brazilian regions, especially taking to account a financial crisis context with low investment in patrolling and inspection process by the government, and also, the illegal trafficking income represents an alternative profit for some wild bird traders. In view of this perspective, it is recommended more population studies regarding the bird species explored, educational policies aiming to highlights the various negative implications of raising animals as pets, and greater strategies against illegal trade in the region. Environmental education programmes should be directed to individuals involved in the practice of raising and trading wild birds, but also to students in schools and to the general public, through press and broadcast media.

## Conclusions

The rich diversity of songbird species that are marketed and kept as pets in the study region reflects the availability of and easy access to these animals as well as their economic and cultural importance. However, the number of species mentioned did not vary with the income, level of education, or age of the respondents. It has been shown that admiration and appreciation for singing are the main reasons for the local exploitation and trade of birds. Due to their small size, ease of maintenance, and high singing capacity, species of the family Thraupidae have the highest UVs, and species endemic to the Caatinga biome, such as *S*. *albogularis* and *P*. *dominicana*, suffer greater pressure from illegal trade and captive bird-keeping. This family had the highest UVs among the species reported in this study, indicating that the populations of each of these species are at risk due to removal from their natural environment.

Whether purchased or captured, respondents prefer male birds since they have the greatest capacity for singing, so male individuals suffer greater pressure from use. The results also indicate that males can be trained to improve the quality of their singing, consequently increasing their commercial value, but maintaining these birds in cages requires substantial care to maintain their health and song quality. Another important use is the exploitation of the Saffron Finch, *S*. *flaveola*, for fighting (known locally as “rinhas”), a clandestine activity that provides entertainment and potential financial returns for people who place bets on the fights.

Although considered illegal in Brazil, the keeping and sale of songbirds among people of different ages is common in the semi-arid region of the country. In this context, ethnoornithological studies are fundamentally important since they can provide basic information to inform plans and actions for conservation and sustainable management of the local avifauna, including environmental education strategies as an essential element.

## Abbreviations

CAAE: Certificate of Presentation for Ethical Consideration
CBRO: Brazilian Ornithological Records Committee
CETAS: Wild Animal Triage Centre
HDI: Human development index
HULW: Hospital Universitário Lauro Wanderley
IBAMA: Brazilian Institute of the Environment and Renewable Natural Resources
IUCN: International Union for Conservation of Nature
MMA: Ministry of the Environment
PAST: Paleontological Statistics
UV: Use value
